# In-silico performance, validation, and modeling of the Nanostring Banff Human Organ transplant gene panel using archival data from human kidney transplants

**DOI:** 10.1186/s12920-021-00891-5

**Published:** 2021-03-19

**Authors:** R. N. Smith

**Affiliations:** Department of Pathology, Massachusetts General Hospital, 501 Warren Bldg, 55 Fruit Street, Boston, MA 02114 USA

**Keywords:** Kidney, Renal, Transplantation, Gene expression, Statistics, Modelling, Classification, Nanostring, BHOT

## Abstract

**Background:**

RNA gene expression of renal transplantation biopsies is commonly used to identify the immunological patterns of graft rejection. Mostly done with microarrays, seminal findings defined the patterns of gene sets associated with rejection and non-rejection kidney allograft diagnoses. To make gene expression more accessible, the Molecular Diagnostics Working Group of the Banff Foundation for Allograft Pathology and NanoString Technologies partnered to create the Banff Human Organ Transplant Panel (BHOT), a gene panel set of 770 genes as a surrogate for microarrays (~ 50,000 genes). The advantage of this platform is that gene expressions are quantifiable on formalin fixed and paraffin embedded archival tissue samples, making gene expression analyses more accessible. The purpose of this report is to test in silico the utility of the BHOT panel as a surrogate for microarrays on archival microarray data and test the performance of the modelled BHOT data.

**Methods:**

BHOT genes as a subset of genes from downloaded archival public microarray data on human renal allograft gene expression were analyzed and modelled by a variety of statistical methods.

**Results:**

Three methods of parsing genes verify that the BHOT panel readily identifies renal rejection and non-rejection diagnoses using in silico statistical analyses of seminal archival databases. Multiple modelling algorithms show a highly variable pattern of misclassifications per sample, either between differently constructed principal components or between modelling algorithms. The misclassifications are related to the gene expression heterogeneity within a given diagnosis because clustering the data into 9 groups modelled with fewer misclassifications.

**Conclusion:**

This report supports using the Banff Human Organ Transplant Panel for gene expression of human renal allografts as a surrogate for microarrays on archival tissue. The data modelled satisfactorily with aggregate diagnoses although with limited per sample accuracy and, thereby, reflects and confirms the modelling complexity and the challenges of modelling gene expression as previously reported.

**Supplementary Information:**

The online version contains supplementary material available at 10.1186/s12920-021-00891-5.

## Background

RNA gene expression is now commonly used to find diagnostic patterns of gene expression in renal transplants. Mostly done using microarrays on fresh tissue, many informative and seminal studies identified the dominant pattern of differential gene expressions associated with renal transplant diagnoses [1–9].

Recent technology, NanoString nCounter, employs formalin fixed paraffin embedded archival tissue as the RNA source for gene expression [20]. To promote gene expression in renal transplants, the Molecular Diagnostic Working Group of the Banff Foundation for Allograft Pathology and NanoString Technologies partnered to create a subset of microarray genes, the Banff Human Organ Transplant (BHOT) panel to encourage more widespread usage of gene expression in allografts [21]. NanoString gene panels employ only 770 gene targets and, therefore, are not gene discovery tools.

Validation of the BHOT panel is best done by comparing the BHOT panel and microarrays on the same RNA, but such an experiment has not yet been done. The purpose of this report is to test in silico if the BHOT panel as a subset of microarray genes shows similar microarray expression patterns as archival microarray data [1–9, 22], with the caveat that some variation in patterns may occur in BHOT vs microarray expression. In addition, modelling studies were performed to test how well the BHOT gene subset identifies the annotated diagnostic classes and, additionally, highlights the practical issues investigators will find when using classification of gene expression for clinical decision making.

## Methods

Abbreviations and their Definitions: See abbreviations under declarations below at the end of this document.

BHOT Panel Genes: [23]

Annotations for Definitions of Pathways and Cell Types: CIBERSORT [24], KEGG [25], Human Blood Atlas [26], BHOT [23].

### Data

Downloaded text files of GSE data sets 30718 [6], 36059 [10, 27, 28], and 48581 [10, 29] from NCBI all derived from HU-133 plus 2 microarrays with their diagnostic annotations were first imported into excel. These three databases established the gene expression patterns for T Cell Mediated Rejection (TCMR), Antibody Mediated Rejection (ABMR), and delayed graft function (Acute Kidney Injury, AKI) [6, 10, 27–29]. These data were joined with the BHOT panel excluding non-BHOT genes and non-renal parenchymal and viral genes. Data were renormalized using the housekeeping probes with negligible effect. Data were then log_2_ transformed.

### Software

Analyses were performed using SAS/JMP 14.2/R4.0.2/JMP Genomics 9.2 using linear models with validation, principal components, multiple logistic regression, K-means clustering, or one-way anova with the Steele-Dwass post-hoc test, which is a non-parametric version of Tukey–Kramer with the addition of an adjusted P value, or Python 3.7 with the sklearn module (Pycaret 2.0), which was used also for multiple classifications. Principal components were robust to suppress outliers. Bayesia Labs 9.0 was used to construct Bayesian Networks. Classification parameters for models are in Additional file 1: Table S1. From power calculations (power > 0.8, usually > 0.9), significance was set at a False Discovery Rate Adjusted *P* value of 0.005 (− log10 = 2.3). This was also applied to the significance of any mean difference. Graphing was performed with Graph Builder (JMP 14.2) or Python 3.7 with matplotlib/seaborn.

Batch effects. UMAP (Uniform Manifold Approximation and Projection for Dimension Reduction) clustering was performed to identify by clustering unknown anomalous effects (batch effects) in the archival data using genes with a coefficient of variation (CV) of ≤ 5%, which included the house keeping genes from BHOT. Genes with CV ≤ 5% have little partitioning value. The three clusters were manually coded as categorical variables, and batch normalization was performed using the lowest CV genes. The lowest 5% CV (coefficient of variations) including housekeeping genes were deleted, leaving 667 genes and 764 samples [30].

### Parsing of genes

Three methods of parsing genes were used to create multiple principal components that were used to partition the diagnostic groups. Principal component analysis was chosen for data reduction due to the massive collinearity of individual gene expressions.

The first method, supervised, finds the highest gene expressions by ANOVA/linear models between two groups, TCMR, ABMR, AKI, MIXED, or NORMAL as compared to NO REJECTION. Multiple principal components with eigen values from 3 to > 100 were derived from each binary comparison. These principal components are called Pathological Based Principal Components (PBPC) [1–9, 31]. The second method, semi-supervised, used genes from CIBERSORT LM22, Blood Atlas, and KEGG, and NanoString annotations to create PCs for a specific cell type or immunological pathway. This method was inspired by Nanostring Advanced Analysis software, in which “scores” are created using singular value decomposition, a sparse principal component, of genes that identify a cell type or immunological pathway. The genes within a cell type or pathway created one principal component with an eigen value > 5 and are called Cell Pathway Principal Components (CPPC). The third method, unsupervised, derives multiple principal components with eigen values from 3 to > 100, from all genes without regard to a class or diagnosis and are called Unsupervised Principal Components (UPC).

### Pathological diagnoses

Pathological diagnoses, derived from annotations of the downloaded databases, are categorical classes: T Cell Mediated Rejection (TCMR), Antibody Mediated Rejection (ABMR), Mixed (both TCMR and ABMR), NO REJECTION (NR), Acute Kidney Injury (AKI) which is defined as renal dysfunction unrelated to rejection and often occurring post transplantation, and Normal Native (NORMAL). These diagnostic classes are summary classes derived from the more complex classifications of renal allograft rejections per Banff classification schemes, which employ microscopic criteria, many of which cannot be evaluated, identified, or correlated with RNA expression [32, 33].

## Results

**Fig. 1:**
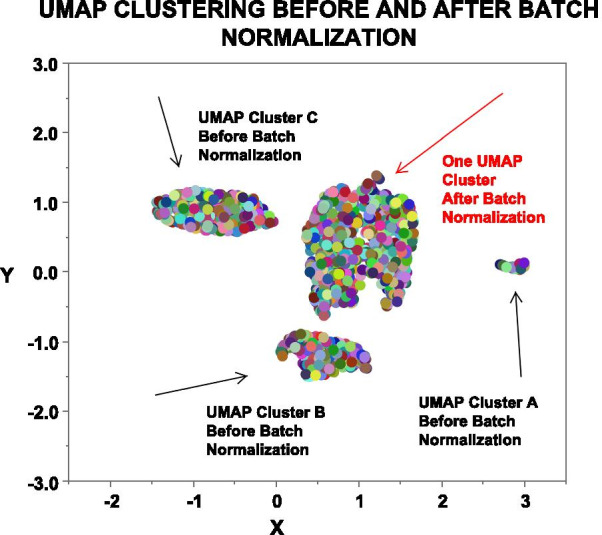
UMAP Clustering. UMAP clustering (JMP 14.2/R4.0.2) of data using genes with a coefficient of variation (CV) of ≤ 5%, which included the house keeping genes identified three groups, A, B, & C, each indicated by a black arrow. After batch normalization in JMP Genomics 9.2, repeat UMAP clustering created one group, red arrow. Each differently colored circle is one sample

Although the combined data were derived from the same array, unknown batch effects can often skew data. It is unknown how many experiments were done to create the archived datasets, so that batch corrections cannot be done on individual experiments. To work around this problem UMAP clustering was performed on the genes with the lowest 5% coefficient of variation. Figure 1 shows a graph in which three clusters were identified with the lowest expressing genes before batch correction. After batch normalization, one cluster remained. Such batch effects have a slight influence (F = 0.02) on classification accuracy (Pycaret classification, compare models module), Additional file 2: Table S2, using all data and the target as DX (diagnosis). Batch normalized data was throughout.

**Fig. 2:**
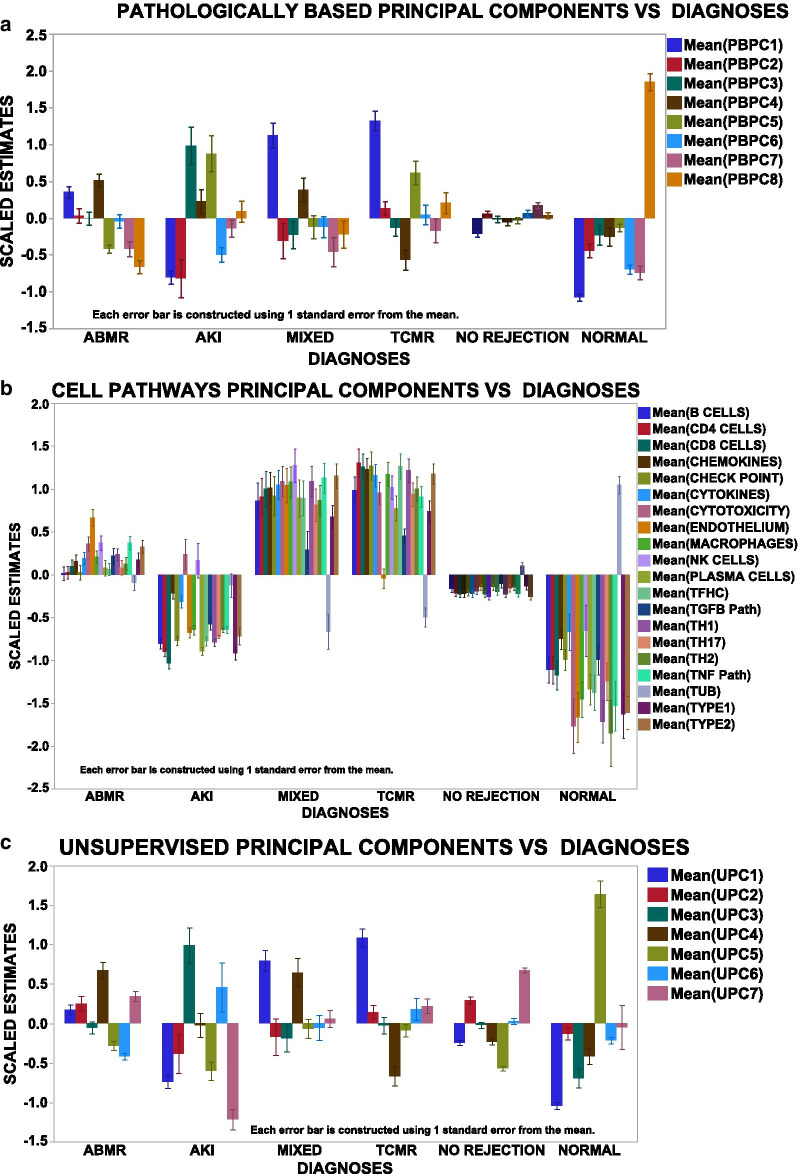
Scaled estimates vs Diagnosis for each of the three types of derived principal components: **a** Pathologically Based (PBPC); **b** Cell Pathways (CPPC); and **c** unsupervised (UPC). Error bar is one SEM. Graph builder JMP 14.2

To find the highest partitioning values (feature selection) of the PCs with the strongest associations with the diagnostic groups, linear models and active effects in multinomial logistic regression were used, confirmed by Pycaret regressions their estimates and significance appear in Additional file 3: Table S3. Graphically, Fig. 2 shows the principal components (PCs) vs DX for the three different methods of gene selection. Pathologically based PCs (PBPCs, Fig. 2a) and the unsupervised PCs (UPC, Fig. 2c) readily partition the diagnostic groups. CPPC (Fig. 2b) identify immunologically interpretable patterns with a high PC for tubules in NORMALS, and a high endothelial PC in ABMR and MIXED but low in the other groups. Inflammatory cell types and mediators are highest in TCMR and MIXED rejections, known to contain inflammatory infiltrates, and low in NORMALS and AKI.

To understand how the PCs distribute among the DX, kernel density estimates appear in S. Fig. 1. The relatively normally distributed PCs are in S. Fig. 1A. S. Figure 1B, C, and D shows average distributions of the PCs for the PBPC, CPPC, & UPC per diagnostic group. The PBPC (1B) shows flat distributions of the diagnoses other than normal, which raises a caveat for their usefulness partitioning diagnostic groups. The CPPC (1C) shows wide separation of the diagnostic groups. The UPC (1D) also shows good separation of the diagnostic groups.

The highest gene expressions per principal component (UPC and PBPC) defined by PC loading tables and confirmed by Partial Least Squares were compared to the transcript patterns previously identified in microarrays called pathologically based transcripts (PBTs) [1–9, 22]. PBPC1 is dominated by genes identifying adaptive immunity, chemokine and cytokine signaling, cytotoxicity, T cell receptor signaling, toll-like receptor signaling, type 2 interferon, CD4 and CD8 T cells, and macrophages and found in PBTs (Type 2 interferon induced, cytotoxic lymphocyte induced, T cell, injury and injury repair transcripts). PBPC1, therefore, is an inflammatory signature that is associated with the inflammation commonly seen in TCMR and MIXED rejections. PBPC2, low in the AKI diagnoses, is low for genes in the cytokine signaling (JAK2) pathway, innate immunity, TH17 pathways, and toll-like receptor signaling and found in PBTs (late injury repair and type 2 interferon induced transcripts). PBPC3, high in AKI, identifies higher and different cytokines (CXCL13, 16, and CXCR6) and is found in PBTs (injury repair, endothelial, type 2 interferon induced, and decreased solute carrier transcripts). PBPC4, highest in ABMR and MIXED rejections, is dominated by the expression of endothelial genes and some CD4 cells and found in PBTs (endothelial and alloantibody induced transcripts), and is an endothelial pattern closely associated with antibody mediated rejections (ABMR and MIXED). PBPC5, high in AKI and TCMR, contains genes for B cells, complement, and innate immunity and is found in PBTs (B cell, macrophage, injury-related transcripts). PBPC6, lowest in the AKI and normal diagnoses is low for genes in innate immunity, type 2 interferon, and CD4s, and CD8 T cells and low in PBTs (injury related type 2 interferon inducible, T cell transcripts). PBPC7, is low for chemokine, T, B endothelial, and macrophage genes and low in PBTs (B cell, alloantibody induced, endothelial injury repair and type 2 interferon induced transcripts). PCPC8, highest in the normal diagnosis is high for glomerular, tubular, TH17 pathway, and tissue homeostasis genes and found in PBTs (solute carrier (high), alloantibody induced (low), endothelial (low), type 2 interferon induced (low)).

Within the unsupervised principal components UPC1, like PBPC1, identifies an inflammatory pattern highest in genes for adaptive immunity, chemokines, cytokines, cytotoxicity, innate immunity, toll-like receptor signaling, CD4 and CD8 T cells, and macrophages. UPC2, highest in NO REJECTION contains type 1 and 2 interferon related gene expressions, chemokine and cytokine, innate and toll-like receptor related gene expressions without inflammatory cells and found in PBT interferon related transcripts. UPC3, highest in AKI, shows the highest gene patterns in cytokines, complement, innate immunity, oxidative stress, without markers for B, T, or macrophage cells and is found in PBTs (interferon and injury repair transcripts and solute carrier (low)). UPC4, high in ABMR and MIXED, identifies an endothelial pattern with many endothelial genes, adaptive immunity, chemokines, complement, cytokines, B cell, CD4, CD8, macrophage genes without any cytotoxicity signals and is found in PBTs (endothelial and alloantibody induced, B cell, type 2 interferon). UPC5, highest in the normal diagnosis includes gene signals for glomeruli, tubules, some innate, oxidative, TH17, TNF without type 2 interferon, plasma, CD4, CD8, or macrophage cells and is found in PBTs (high tubular, high endothelial, injury repair). UPC6, high in AKI, shows the greatest number of genes in adaptive, cytokine, complement, innate, and CD8, and macrophages and is found in PBTs (type 2 interferon, injury related, macrophage related transcripts). UPC7, highest in NO REJECTION, is highest for genes in chemokine, cytokine, innate, oxidative, TH17 pathways, with some markers for B, CD4, CD8, and macrophage cells and is found in PBTs (injury related, type 2 interferon related transcripts).

All the genes within these principal components (PBPC, UPC, and CPPC) are described in many prior publications on gene expression in renal allografts [1–9, 22], confirming that the BHOT panel is a suitable substitute for microarrays.

### Modelling

**Fig. 3:**
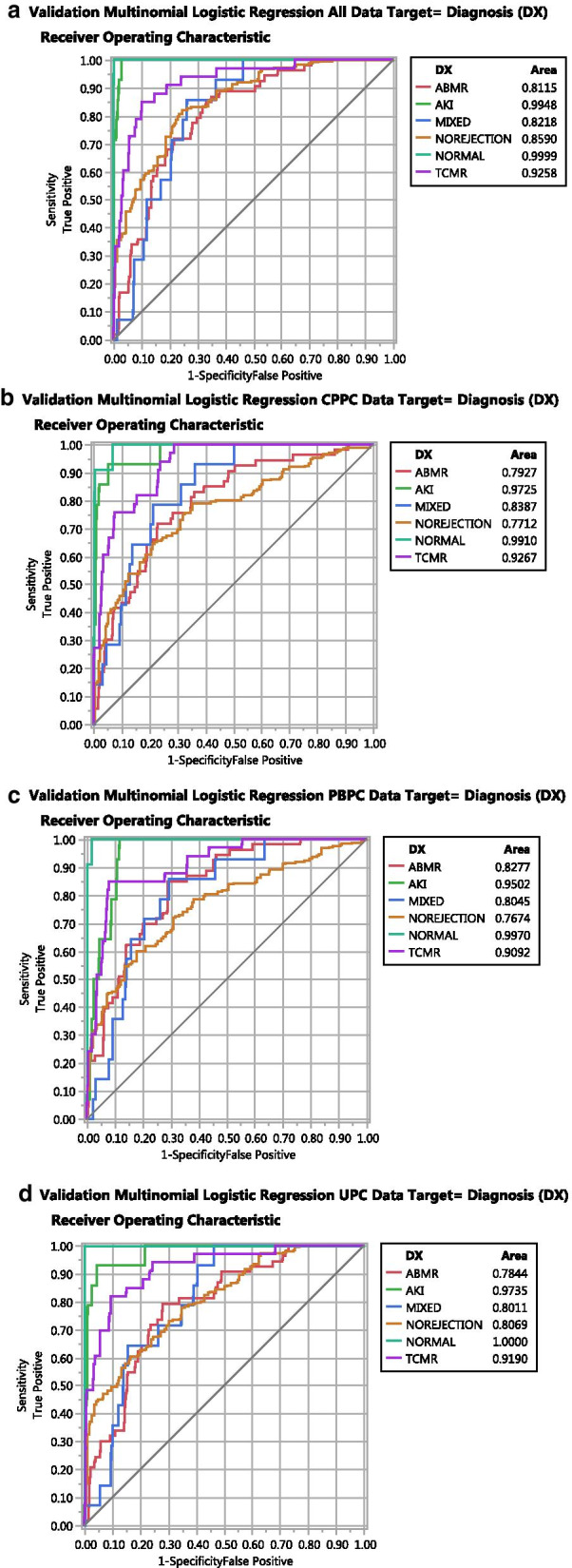
ROC graphs of validated multinomial logistic regression (General Regression, JMP14.2 with adaptive elastic net and K = tenfold validation. Target is diagnosis (DX). **a** Data = All PC (CPPC, PBPC, & UPC); **b** Data = CPPC; **c** Data = PBPC; **d** Data = UPC

Modelling is used to estimate how good variables describe classification parameters, i.e., how accurately the PCs identify the diagnoses. Modelling programs assign a class or in this case a diagnosis to each sample based on the highest probability within the target diagnosis for a specific sample. Initially, multinomial logistic regression modelled the data (CPPC, PBPC, UPC and all three (ALL)) with the target as the diagnosis (DX). All four models created acceptable ROC curves, Fig. 3a–d. The NORMAL and the AKI groups model best because their gene expression patterns are so dramatically different than the rejection groups, S. Fig. 1. The ABMR and NO REJECTION groups model less well. However, the errors in the confusion matrices, a sensitive and easily interpreted classification metric, were substantial, 30–40%, indicating that modelling the genes poorly matched many DX, Table 1A. Even more problematic, the per sample error of 46.9% indicates per sample discrepancies with different types of data engender different patterns of errors. Reeve et.al. also, identified variations in misclassifications when clustering using archetypal analysis as compared to annotated diagnoses [34]. Individual samples are misclassified differently depending on the data set or the modelling algorithm.

**Table 1:** Summary of errors in confusion matrices

Data	Target	Percent errors	Percent errors per sample
*A*			
All	DX	32.8	46.9
CPPC	DX	38	
PBPC	DX	39.6	
UPC	DX	34	
*B*			
All	Clusters	5.9	27.9
CPPC	Clusters	10.4	
PBPC	Clusters	8.6	
UPC	Clusters	16.6	

Percent Errors = Percent Errors from Confusion Matrix

Percent Sample Errors = Percent of Samples with a Single Discordance between Confusion Matrices of a Target per Data Group, Average of Four Data Groups

From Multinomial Logistic Regression, General Regression Model, JMP 14.2. Regression parameters in Supplemental Table 1

**Fig. 4:**
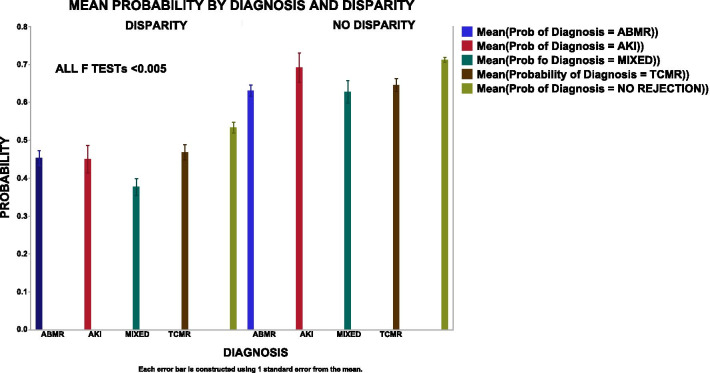
Mean Probability vs Disparity. Using the regression in 3A, the regression creates a column for the probability for each of the six diagnoses for each sample. The highest probability is assigned as most likely DX. The disparities between the annotated DX and the most likely DX creates the confusion matrix. Left = Disparity: DX does not equal most likely DX. Right = No disparity: DX = most likely DX

Although the ROC curves suggest workable models, the misclassifications in the error matrices are excessive for clinical decision making. The classification assignment for the error matrices is derived from the highest probability per group, whether the highest probability is below or above 50%. The average probabilities were examined in misclassified and concordant samples, Fig. 4. Misclassified samples have lower average probabilities (left, disparities) as compared to concordant samples (right, concordant) suggesting that many samples without a consistently high probability cause per sample variations in the error matrices [34].

**Fig. 5:**
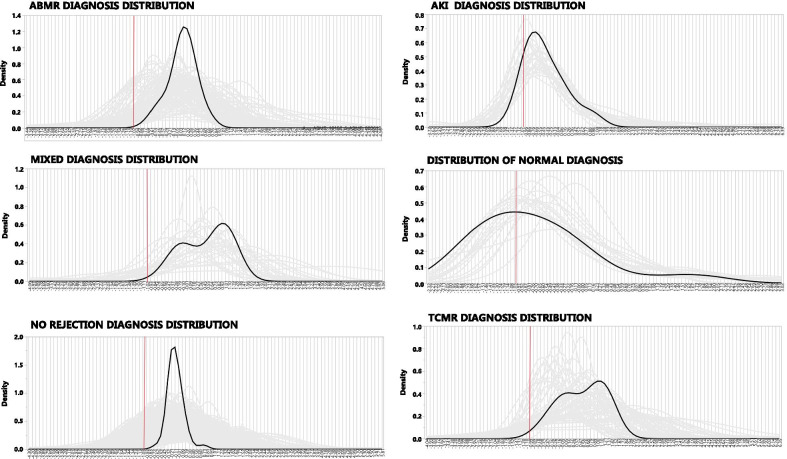
Distribution of all PC (CPPC, PBPC, & UPC) for each of the six annotated diagnoses (JMP Genomics 9.2/Distribution). Grey lines are individual samples. Black curve is the average. The red vertical line is the average of normal for reference

Two possibilities exit for the per sample misclassification patterns: (1) The annotated diagnosis is not a pure category or that (2) Gene expression heterogeneity exists within samples of a diagnostic class. To explore this, distributions were analyzed for all principal components by diagnosis. Figure 5 shows the distributions of all the principal components from the three data sets (CPPC, UPC, and PBPC) for the six diagnostic classes for each sample (grey lines) and a group mean (black line). The grey lines show wide distribution patterns within a diagnosis, and the group mean shows biphasic distributions for the diagnoses AKI, TCMR, and MIXED, which are, therefore, mixtures of distributions. The red vertical line is the average of NORMAL for reference. Vast heterogeneity is evident for all diagnoses.

To test if a better model could be created by reducing the heterogeneity across samples, the samples were clustered using K Means, resulting in 9 clusters by optimal cubic clustering criterion. Using multinomial logistic regression with all PCs and the 9 clusters as the target regression created good models. Table 1B shows that the percent misclassification from the confusion matrices dropped to 6–17%, a dramatic improvement as compared to using the DX as the target, Table 1A. However, the per sample errors in misclassifications were 27.9%, which were much lower than 46.9% using the DX as the target. Table 2 is a contingency table of DX vs clusters. NORMAL cluster and Cluster 6 are similar, but the other diagnoses are widely distributed among the clusters, especially NO REJECTION. These findings suggest that gene expression heterogeneity within the diagnoses and/or impurity of the diagnosis as a categorical variable are contributors to suboptimal classifications.

**Table 2:** Contingency table of diagnoses (DX) vs Clusters

DX/clusters	1	2	3	4	5	6	7	8	9	Total responses
ABMR	0	6	18	4	49	0	13	8	7	105
AKI	4	5	8	0	0	0	0	11	0	28
Mixed	0	2	0	6	12	0	6	2	0	28
TCMR	4	0	7	24	3	0	13	11	5	67
Norejection	25	31	137	20	39	1	98	55	108	514
Normal	0	0	0	0	0	22	0	0	0	22

To determine if other modelling algorithms might improve and/or confirm the prior models, Pycaret, which uses Python and sklearn modules, was used to test additional models, Additional file 1: Table S1 for parameters. The Pycaret permits comparison of multiple models to find the most optimal model by accuracy. The models tested and their parameters are found in Table 3. The best model with the highest accuracy was also tested with the tuning, bagging, boosting, and blending with negligible improvements in accuracy (data not shown). Making an ensemble model of the top three models also did not show any improvement in accuracy. Three models for the data (All, CPPC, PBPC, UPC) with targets as DX and Clusters is shown in Table 3A (DX) and Table 3B (Clusters) with Clusters showing fewer discrepancies. Modeling individual sets (CPPC, PBPC, and UPC) vs the target DX showed a range of confusion matrix errors of 25 – 37%, Table 3C. Per sample discrepancies appear in Table 3D (DX) and Table 3E (Clusters) with Clusters showing fewer discrepancies. With PyCaret (Table 3F and G) showed similar patterns with dramatic improvements in accuracies (> 0.9) using Clusters as the target and with dramatic reductions in the error rates (6–7%).

**Table 3:** Additional models for confusion matrices/discrepancies

*A. Average discrepancies between Diagnoses All PC*					
Model	Target	Data	% Discrepancies		
Multinomial logistic regression	Diagnoses	ALL PC	37.3		
Boot strap forest	Diagnoses	ALL PC	24.1		
Partial least squares	Diagnoses	ALL PC	37.3		

*B. Average discrepancies between Clusters All PC*					
Model	Target	Data	% Discrepancies		
Multinomial logistic regression	Clusters	ALL PC	10.0		
Boot strap forest	Clusters	ALL PC	12.2		
Partial least squares	Clusters	ALL PC	15.1		

*C. Average discrepancies CPPC, UPC, & PBPC*					
Model	Target	Data	% Discrepancies		
Multinomial logistic regression	Diagnoses	CPPC	36.9		
Boot strap forest	Diagnoses	CPPC	25.6		
Partial least squares	Diagnoses	CPPC	27.2		
Multinomial logistic regression	Diagnoses	UPC	24.4		
Boot strap forest	Diagnoses	UPC	24.9		
Partial least squares	Diagnoses	UPC	27.0		
Multinomial logistic regression	Diagnoses	PBPC	35.2		
Boot strap forest	Diagnoses	PBPC	27.5		
Partial least squares	Diagnoses	PBPC	29.2		

*D. Average per sample discrepancies between Diagnoses All PC*					
Model	Target	Data	% Discrepancies		
Multinomial logistic regression vs Boot strap forest	Diagnoses	ALL PC	33.1		
Multinomial logistic regression vs Partial least squares	Diagnoses	ALL PC	32.7		
Boot strap forest vs Partial least squares	Diagnoses	ALL PC	33.1		

*E. Average per sample discrepancies between Clusters All PC*					
Model	Target	Data	% Discrepancies		
Multinomial logistic regression vs Boot strap forest	Clusters	ALL PC	12.4		
Multinomial logistic regression vs Partial least squares	Clusters	ALL PC	13.5		
Boot strap forest vs Partial least squares	Clusters	ALL PC	14.4		

F. Additional models for diagnoses all PC, CPPC, PBPC, & UPC*					
Model	Target	Data	Mean accuracy	Average precision/recall	Percent errors
Extreme Gradient Boosting	Diagnoses	ALL PC	0.74	0.79	36.5
Linear Discriminant Analysis	Diagnoses	CPPC	0.75	0.78	32.6
Extra Tree Classifier	Diagnoses	PBPC	0.75	0.76	35.3
Linear discriminant analysis	Diagnoses	UPC	0.76	0.70	33.9

G. Additional models for clusters all PC, CPPC, PBPC, & UPC*					
Model	Target	Data	Mean accuracy	Average precision/recall	Percent errors
Multinomial logistic regression	Clusters	ALL PC	0.93	0.95	6.9
Extra tree classifier	Clusters	CPPC	0.90	0.93	6.9
Extreme gradient boosting	Clusters	PBPC	0.92	0.94	6.2
Multinomial logistic regression	Clusters	UPC	0.92	0.94	6.9

*Best model chosen; N = 12

A Bayesian Network Model using CPPC was also created, Additional file 6: Figure S2 and Additional file 4: Table S4, because some data are non-parametric, (Fig. 5, parameters in Additional file 1: Table S1). Graph Additional file 6: Figure S2A and B show a graphical network with the center node as the target (DX or Clusters) using the CPPC. Optimal binning algorithms create a high or low value as a categorical variable for each CPPC. Arrows indicate the complex interdependent relationships among the variables as determined by their mutual information/Kulback-Leibler divergence and show the complex conditional probabilities of the variables including the categorical targets (DX or Cluster). S. Figure 2C and D show the Bayes factors (BFs, definition in Abbreviations) for DX and Clusters. For DX (S. Fig. 2C) NORMAL is high for the TUB PC and low for the other CPPC, the expected finding. ABMR, MIXED, and TCMR show high inflammatory CPPC with TCMR showing low ENDO. These BF patterns are like those in Fig. 2. Most notable are the low BFs for NO REJECTION, any demonstrating the difficulty of resolving this heterogeneous group. S. Figure 2D shows the BFs for the Clusters with Cluster 6 resembling the NORMAL DX. The other Clusters show inflammatory CPPC with variations in ENDO and TUB, again showing the complex patterns of gene expressions and the difficulty of resolving the Clusters into known diagnostic clinically useful groups, Table 2. Additional file 4: Table S4A shows the percent error with this model is high 56% for DX with a poor mean ROC of 77% (underlined) and a high log-loss. Again, the Clusters show improved mean ROC 91% and fewer discrepancies (34%), both underlined and with a lower log-loss, again suggesting that clustering creates a simpler model, Additional file 4: Table S4B.

## Discussion

Findings show that the BHOT panel of genes recapitulates the diagnostic patterns identified in seminal archival data, using either of three methods of parsing the genes into principal components (Fig. 2, Fig. 5, Additional file 3: Table 3). As the selected BHOT panel genes are derived partially from many microarray studies, it is not surprising that BHOT panel genes identify the expected patterns of rejection. All three methods of parsing genes created workable models with high average ROC scores. It is unclear which method of parsing the genes into principal components is easiest or most suitable to create an efficient and standardized data analysis pipeline. Using PCs (PBPC) from sets of the highest genes between binary diagnostic comparisons is conceptually simple but engenders many principal components, which share collinearity and make feature selection for modelling both tedious and difficult. Using principal components of cell types and pathways is conceptually easier to understand immunologically. Creating unsupervised principal components is the easiest for feature selection and has an advantage that a latent variable or pathway may be present, which is not readily identified by the first two gene selection methods [18, 19]. These three methods, including just finding the highest genes by t-tests, will likely vary between independently derived data sets because results are very dependent on the sample size of the data set, the balance of the classes employed, and the purity of the annotated class diagnosis.

Some investigators argue that gene expression models assign a more accurate diagnosis than the original diagnosis, and use such models for clinical diagnosis. However, heterogeneity is present in the misclassification assignments per sample by different models [34] or data (CPPC, UPC, PBPC), (Tables 1 and 3, Additional file 4: Table S4). As different modelling algorithms or slightly different PC sets engender inconsistent discordant patterns of misclassifications, changes to the sample diagnosis by modelling may be premature. Model averaging or an ensemble of models does not solve this problem as a new error matrix is created, which still maintains the per sample variations in classifications. Arbitrarily using a high probability to assign a diagnostic classification solves part of the misclassification problem by reducing some misclassifications, but many samples could remain unclassified [34]. To improve assignment of diagnoses of ambiguously modelled samples, additional clinical information such as histological parameters, alloantibody, C4d, or time after transplant, all of which use expert knowledge of prior probabilities, could be incorporated with gene expression to create a clinical pathological diagnosis [34, 35]. Although using expert knowledge may allow assignment of some samples to canonical or variations of canonical diagnoses, and make overall interpretation easier, interpreting such heterogeneous variables, absent in the model, is subjectively biased and may work for some samples but not all.

Although clustering data independent of diagnosis makes a better model with fewer misclassifications, interpreting synthetic clusters remains problematic. It is better to find the best model for the data rather than find the best data for a model. Are these synthetic clusters just “toy” data, that models well but has no biological relevance? Some clusters resemble canonical diagnoses, but others do not. How do you assign a meaningful and clinically interpretable name to synthetic cluster? Nevertheless, creating more homogeneous groups of samples from clustered data may identify clinically important subgroups, not appreciated in the annotated classes [19, 31]. This is most important in the NO REJECTION diagnosis, which is the most heterogeneous by gene expression (Tables 2 and 3, Additional file 4: Table S4) and the most frequent diagnosis. This diagnosis lacks evidence of rejection, and subjects usually have a preserved creatinine, yet the gene expression pattern within the NO REJECTION diagnosis is markedly heterogeneous. If some gene expression subset patterns within the NO REJECTION diagnostic category correlate with a subsequent clinical rejection or correlate with renal outcome, then analysis of gene expressions within clusters or class subsets adds value to clinical and pathological decisions.

The gene expression data are heterogeneous within the original diagnostic classes because clustering all the principal components creates a better model with fewer misclassifications. This is most likely because pathological diagnoses are complex and critically dependent on microscopic findings that cannot be identified within a mixture of extracted RNAs. For example, tubulitis, which is mononuclear inflammation identified within tubules, is required for a diagnosis of TCMR but cannot be captured in a slurry of RNA. In addition, many of the Banff histological lesions used or required for allograft diagnoses are also somewhat non-specific [10, 27, 36]. Additionally, the rejection classes (TCMR, ABMR, and MIXED) used to categorize the data are summaries of rejections patterns, which have grades from clinically mild to clinically very serious, so that the annotated class diagnoses are heterogeneous and mere summary approximations. Clustering gene expression data may find clinical subsets not appreciated within the original annotated diagnoses.

Modelling creates challenges for investigators, who wish to use gene expression to inform diagnostic decisions because classifications do not uniformly assign a consistent diagnosis per sample. This is problematic as investigators using different models or slightly differently derived data sets could reassign diagnoses discordantly. Uncertainties could also arise in clinical trials using allograft transplant gene expression for classifications if contributors to the clinical trial assign variant gene expression classifications, depending on how the genes are analyzed or modelled. This problem also applies to aligning disparate studies investigating a similar hypothesis. Additional information as covariates might include creatinine trajectory, proteinuria, time after transplant, prior diagnoses, or histological parameters might improve or alter modelling performance [37, 38].

Although clustering the data independent of the annotated DX makes modelling more consistent and lowers misclassification rates, it is unclear if these additional categories represent biologically relevant diagnostic classes, inconsequential minor variants, biopsy sampling error or evolving forms of established diagnoses. Only correlation of pathological diagnoses and gene expression patterns with the endpoint of renal allograft survival or subsequent rejections can resolve such discrepancies and identify the optimal and biologically relevant classes for clinical decision-making. This is likely best done by longitudinal analysis of a patients’ samples.

## Conclusion

These findings confirm the BHOT gene panel is a suitable surrogate for microarrays and identifies the expected patterns in human allografts. These findings also confirm the complexity of modelling gene expressions and suggest that reassigning a diagnosis based solely on gene expression is not straightforward for clinical decision making. Future analytical challenges facing investigators include: (1) how and which genes are best and most efficiently parsed to create an efficient data analysis pipeline; (2) how is best modelling performed to assign a diagnosis to a patient’s sample; (3) what clinical and pathological parameters improve model performance; (4) how to resolve the heterogeneity of gene expression and pathological diagnoses into more homogeneous groups that permit the most accurate modelling and immunological interpretation; and finally, (5) determine if new and more homogeneous classes are biologically relevant.

## Supplementary Information

**Additional file 1**. **Supplementary Table 1**. Classification Parameters

**Additional file 2**. **Supplementary Table 2**. Classification Accuracies Before and After Batch Normalization

**Additional file 3**. **Supplementary Table 3**. Estimates and -Log10 False Discovery Rate of PV for the Principal Components and Pathways vs Diagnoses

**Additional file 4**. **Supplementary Table 4**. Error Matrices from Classification of Bayesian Networks

**Additional file 5**. **Figure 1** Kernel Density Estimates of Principal Components (JMP Genomics9.2/Distribution). 1A. Kernel density estimates, all PCs. 1B-1D. Kernel density estimates of PBCP (1B), CPPC (1C), and UPC (1D) by Diagnosis.

**Additional file 6**. **Figure 2**. Graphical representation of Bayesian Networks for DX (A) and Cluster (B). Bayesia Lab 9.4. Discretization: Perturbed Tree, Bins = 2; Supervised Learning = Tree Augmented Naïve Bayes, both determined empirically by minimal descriptive length. Bargraphs of the Bayes Factors for DX (C) and Clusters (D) following calculation for Kulback-Leibler divergence

## Data Availability

Source data in public domain: (1) GSE36059: https://www.ncbi.nlm.nih.gov/geo/query/acc.cgi?acc=GSE36059; (2) GSE30718 https://www.ncbi.nlm.nih.gov/geo/query/acc.cgi?acc=GSE30718; (3) GSE48581 https://www.ncbi.nlm.nih.gov/geo/query/acc.cgi?acc=GSE48581. All code and calculations available from author.

## Abbreviations

− 10FDR: Negative LOG10 FDR
ABMR: Antibody mediated rejection
AKI: Acute kidney injury, non-rejection injury
ALL: All three sets of principal components: CPPC, PBPC, UPC
Bayes Factor: BF, posterior probability with data/posterior probability without data
BHOT: Banff Human Organ transplant panel of gene targets
CD4CELLS: Principal components of CD4 T cell genes
CD8CELLS: Principal components of CD8 T cell genes
CHEMO1: Principal components of chemokine genes
Clusters: 9 Clusters by K-means
CP: Principal components of cell T check point inhibitor T cell genes
CPPC: Cell pathway principal components
CV: Coefficient of variation
CYTOK1: Principal components of cytokine genes
CYTOTOX: Principal components of cytotoxicity genes
DX: Annotated diagnosis
ENDO: Principal components of endothelial genes
Fig: Figure
FDR: False discovery rate adjusted *P* value
MACS: Principal components of macrophage genes
Mixed: ABMR and TCMR
NK: Principal components of NK cell genes
No Rejection: No evidence of rejection
Normal: Native kidneys
PBPC: Pathological based principal components
PC: Principal component
PLASMA: Principal components of plasma cell genes
TCMR: T cell mediated rejection
S: Supplemental
TFHC: Principal components of T follicular helper cell genes
TGFB: Principal components of TGFB relative genes
TH1: Principal components of T helper cell type 1 genes
TH17: Principal components of T helper cell 17 genes
TH2: Principal components of T helper cell type 2 genes
TNF: Principal components of TNF related genes
TUB: Principal components of renal tubular epithelial genes
TYPE1: Principal components of Type 1 interferon related genes
TYPE2: Principal components of Type 2 interferon related genes
TYPE2: Principal components of Type 2 interferon related genes
UMAP: Uniform manifold approximation and projection for dimension reduction
UPC: Unsupervised principal components
