# Supplementary material for: In-silico performance, validation, and modeling of the Nanostring Banff Human Organ transplant gene panel using archival data from human kidney transplants
Source: BMC Med Genomics. 2021 Mar 19;14:86. doi: 10.1186/s12920-021-00891-5 (PMC7977303; doi:10.1186/s12920-021-00891-5)
Supplement: Supplementary file 1 — Additional file 1. Supplementary Table 1. Classification Parameters [file 12920_2021_891_MOESM1_ESM.pdf]

SUPPLEMENTARY TABLE 1
CLASSIFICATION PARAMETERS

CLASSIFICATION PARAMETERS

Pycaret Modules	Pycaret Classifiers Tested:
Setup:	Ada Boost Classifier
remove_multicollinearity=True	CatBoost Classifier
multicollinearity_threshold= 0.95	Decision Tree Classifier
feature_selection=True	Extra Trees Classifier
feature_interaction=True	Extreme Gradient Boosting
fix_imbalance=True	Gaussian Process Classifier
K-fold=10 (default)	Gradient Boosting Classifier
Compare_models=Classifiers	K Nearest Neighbour
Create_model	Light Gradient Boosting
Evaluate_model	Linear Discriminant Analysis
	Logistic Regression
	Multi Level Perceptron
	Naives Bayes
	Quadratic Discriminant Analysis
	Random Forest Classifier
	Ridge Classifier
	SVM – Linear Kernel
	SVM – Radial Kernel
**JMP14.2**	**Bayesia Lab 9.0**
General Regression	Discretization Bin =2, Perturbed Tree*
Multinomial Logistic Regression	Supervised Learning = Tree Augmented Naïve Bayes*
Adaptive elastic net, penalty for colinearity	Kulback-Leibler Divergence
K-fold=10	Resampling K Fold =10
Repeat with active effects until no parameters are dropped	*Empirically Determined by Minimal Descriptive Length
