# Supplementary material for: In-silico performance, validation, and modeling of the Nanostring Banff Human Organ transplant gene panel using archival data from human kidney transplants
Source: BMC Med Genomics. 2021 Mar 19;14:86. doi: 10.1186/s12920-021-00891-5 (PMC7977303; doi:10.1186/s12920-021-00891-5)
Supplement: Supplementary file 2 — Additional file 2. Supplementary Table 2. Classification Accuracies Before and After Batch Normalization [file 12920_2021_891_MOESM2_ESM.pdf]

SUPPLEMENTARY TABLE 2

Pre-Batch		Post-Batch	
Model	Accuracy	Model	Accuracy
Linear Discriminant Analysis	0.72	Logistic Regression	0.76
SVM - Linear Kernel	0.72	Linear Discriminant Analysis	0.76
Logistic Regression	0.71	Light Gradient Boosting Machine	0.76
Extreme Gradient Boosting	0.71	Extra Trees Classifier	0.76
Extra Trees Classifier	0.71	Exgtreme Gradnient Boosting	0.76
Light Gradient Boosting Machine	0.70	Ridge Classifier	0.75
Random Forest Classifier	0.70	K Neighbors Classifier	0.75
Gradient Boosting Classifier	0.70	Random Forest Classifier	0.74
Ridge Classifier	0.70	Gradient Boosting Classifier	0.74
K Neighbors Classifier	0.69	SVM - Linear Kernel	0.74
Ada Boost Classifier	0.67	Ada Boost Classifier	0.72
Naïve Bayes	0.55	Naïve Bayes	0.60
