# Supplementary material for: In-silico performance, validation, and modeling of the Nanostring Banff Human Organ transplant gene panel using archival data from human kidney transplants
Source: BMC Med Genomics. 2021 Mar 19;14:86. doi: 10.1186/s12920-021-00891-5 (PMC7977303; doi:10.1186/s12920-021-00891-5)
Supplement: Supplementary file 3 — Additional file 3. Supplementary Table 3. Estimates and -Log10 False Discovery Rate of PV for the Principal Components and Pathways vs Diagnoses [file 12920_2021_891_MOESM3_ESM.pdf]

SUPPLEMENTARY TABLE 3

PC	DIAGNOSIS	Estimate	-LOG10 FDRPV	
B CELLS	ABMR	0.06	0.3	
CD4 CELLS	ABMR	0.03	0.2	
CD8 CELLS	ABMR	0.12	0.9	
CHECK POINT	ABMR	-0.01	0.1	A
CHEMOKINES	ABMR	-0.05	0.3	
CYTOKINES	ABMR	-0.01	0.0	
CYTOTOXICITY	ABMR	0.25	2.4	
ENDOTHELIUM	ABMR	0.81	22.0	
MACROPHAGES	ABMR	0.19	2.1	
NK CELLS	ABMR	0.05	0.2	
PBPC1	ABMR	0.24	3.7	
PBPC2	ABMR	0.26	2.6	
PBPC3	ABMR	-0.07	0.3	
PBPC4	ABMR	0.47	9.5	
PBPC5	ABMR	-0.55	11.6	
PBPC6	ABMR	0.16	1.7	
PBPC7	ABMR	-0.12	0.8	
PBPC8	ABMR	-0.89	28.7	
PLASMA CELLS	ABMR	0.19	1.8	
TFHC	ABMR	0.09	0.6	
TGFB Path	ABMR	0.34	5.6	
TH1	ABMR	0.27	3.7	
TH17	ABMR	0.13	1.1	
TH2	ABMR	0.24	2.2	
TNF Path	ABMR	0.37	6.1	
TUBULES	ABMR	-0.06	0.4	
TYPE1 INTERFERON	ABMR	0.36	5.7	
TYPE2 INTERFERON	ABMR	0.32	5.8	
UPC1	ABMR	0.17	2.9	
UPC2	ABMR	0.25	2.7	
UPC3	ABMR	-0.05	0.3	
UPC4	ABMR	0.67	19.6	
UPC5	ABMR	-0.28	6.8	
UPC6	ABMR	-0.42	6.6	
UPC7	ABMR	0.35	6.4	
B CELLS	AKI	-0.76	23.3	
CD4 CELLS	AKI	-0.90	30.5	
CD8 CELLS	AKI	-1.02	38.6	
CHECK POINT	AKI	-0.81	25.0	
CHEMOKINES	AKI	-0.42	9.0	
CYTOKINES	AKI	-0.51	11.7	
CYTOTOXICITY	AKI	0.12	0.8	
ENDOTHELIUM	AKI	-0.54	10.4	
MACROPHAGES	AKI	-0.66	19.7	
NK CELLS	AKI	-0.15	1.0	
PBPC1	AKI	-0.92	41.3	
PBPC2	AKI	-0.59	10.9	
PBPC3	AKI	0.92	27.7	
PBPC4	AKI	0.18	1.8	
PBPC5	AKI	0.74	19.9	
PBPC6	AKI	-0.29	4.4	
PBPC7	AKI	0.16	1.2	
PBPC8	AKI	-0.13	1.0	
PLASMA CELLS	AKI	-0.78	23.0	
TFHC	AKI	-0.75	22.6	
TGFB Path	AKI	-0.45	9.4	
TH1	AKI	-0.75	23.6	
TH17	AKI	-0.67	18.5	
TH2	AKI	-0.54	9.1	
TNF Path	AKI	-0.64	16.4	
TUBULES	AKI	-0.09	0.6	
TYPE1 INTERFERON	AKI	-0.73	20.4	
TYPE2 INTERFERON	AKI	-0.73	25.8	
UPC1	AKI	-0.74	39.5	
UPC2	AKI	-0.38	5.5	
UPC3	AKI	0.99	38.5	
UPC4	AKI	-0.02	0.1	
UPC5	AKI	-0.60	26.9	
UPC6	AKI	0.46	7.9	
UPC7	AKI	-1.21	60.3	
B CELLS	MIXED	0.90	31.4	
CD4 CELLS	MIXED	0.91	31.3	
CD8 CELLS	MIXED	1.02	38.9	

CHECK POINT	MIXED	0.88	29.2
CHEMOKINES	MIXED	0.81	30.3
CYTOKINES	MIXED	0.85	29.5
CYTOTOXICITY	MIXED	0.98	28.6
ENDOTHELIUM	MIXED	1.18	43.5
MACROPHAGES	MIXED	1.06	45.4
NK CELLS	MIXED	0.96	23.9
PBPC1	MIXED	1.01	48.6
PBPC2	MIXED	-0.08	0.4
PBPC3	MIXED	-0.29	3.4
PBPC4	MIXED	0.34	5.2
PBPC5	MIXED	-0.25	2.8
PBPC6	MIXED	0.09	0.6
PBPC7	MIXED	-0.16	1.3
PBPC8	MIXED	-0.44	8.1
PLASMA CELLS	MIXED	1.01	35.9
TFHC	MIXED	0.92	32.6
TGFB Path	MIXED	0.42	8.0
TH1	MIXED	1.13	48.0
TH17	MIXED	0.87	29.3
TH2	MIXED	0.98	27.6
TNF Path	MIXED	1.13	45.2
TUBULES	MIXED	-0.63	17.4
TYPE1 INTERFERON	MIXED	0.87	27.8
TYPE2 INTERFERON	MIXED	1.15	57.0
UPC1	MIXED	0.79	44.4
UPC2	MIXED	-0.17	1.4
UPC3	MIXED	-0.19	2.0
UPC4	MIXED	0.64	17.9
UPC5	MIXED	-0.07	0.6
UPC6	MIXED	-0.05	0.3
UPC7	MIXED	0.06	0.4
B CELLS	TCMR	1.03	39.5
CD4 CELLS	TCMR	1.31	58.3
CD8 CELLS	TCMR	1.28	57.3
CHECK POINT	TCMR	1.23	52.1
CHEMOKINES	TCMR	1.03	45.9
CYTOKINES	TCMR	0.96	36.5
CYTOTOXICITY	TCMR	0.84	21.7
ENDOTHELIUM	TCMR	0.10	0.6
MACROPHAGES	TCMR	1.16	52.3
NK CELLS	TCMR	0.70	13.5
PBPC1	TCMR	1.21	65.3
PBPC2	TCMR	0.36	4.5
PBPC3	TCMR	-0.19	1.7
PBPC4	TCMR	-0.61	15.7
PBPC5	TCMR	0.48	9.0
PBPC6	TCMR	0.25	3.4
PBPC7	TCMR	0.13	0.9
PBPC8	TCMR	-0.02	0.1
PLASMA CELLS	TCMR	0.89	28.7
TFHC	TCMR	1.29	58.5
TGFB Path	TCMR	0.58	14.5
TH1	TCMR	1.25	57.1
TH17	TCMR	0.99	37.0
TH2	TCMR	1.12	34.7
TNF Path	TCMR	0.91	31.0
TUBULES	TCMR	-0.46	9.8
TYPE1 INTERFERON	TCMR	0.93	31.5
TYPE2 INTERFERON	TCMR	1.17	59.0
UPC1	TCMR	1.08	74.2
UPC2	TCMR	0.15	1.1
UPC3	TCMR	-0.03	0.1
UPC4	TCMR	-0.67	19.3
UPC5	TCMR	-0.09	1.0
UPC6	TCMR	0.18	1.5
UPC7	TCMR	0.22	3.0
B CELLS	NOREJECTION	-0.15	1.4
CD4 CELLS	NOREJECTION	-0.25	3.0
CD8 CELLS	NOREJECTION	-0.24	3.0
CHECK POINT	NOREJECTION	-0.27	3.4
CHEMOKINES	NOREJECTION	-0.42	9.5
CYTOKINES	NOREJECTION	-0.43	8.5
CYTOTOXICITY	NOREJECTION	-0.30	3.4
ENDOTHELIUM	NOREJECTION	-0.02	0.1
MACROPHAGES	NOREJECTION	-0.27	3.9

NK CELLS	NOREJECTION	-0.59	9.9
PBPC1	NOREJECTION	-0.35	7.3
PBPC2	NOREJECTION	0.26	2.5
PBPC3	NOREJECTION	-0.09	0.5
PBPC4	NOREJECTION	-0.07	0.4
PBPC5	NOREJECTION	-0.15	1.2
PBPC6	NOREJECTION	0.28	4.1
PBPC7	NOREJECTION	0.44	7.3
PBPC8	NOREJECTION	-0.15	1.3
PLASMA CELLS	NOREJECTION	-0.07	0.4
TFHC	NOREJECTION	-0.20	2.2
TGFB Path	NOREJECTION	-0.01	0.1
TH1	NOREJECTION	-0.22	2.6
TH17	NOREJECTION	-0.13	1.0
TH2	NOREJECTION	-0.06	0.3
TNF Path	NOREJECTION	-0.24	2.9
TUBULES	NOREJECTION	0.16	1.6
TYPE1 INTERFERON	NOREJECTION	0.02	0.1
TYPE2 INTERFERON	NOREJECTION	-0.29	4.9
UPC1	NOREJECTION	-0.27	6.3
UPC2	NOREJECTION	0.28	3.2
UPC3	NOREJECTION	-0.03	0.2
UPC4	NOREJECTION	-0.21	2.5
UPC5	NOREJECTION	-0.59	26.3
UPC6	NOREJECTION	0.05	0.2
UPC7	NOREJECTION	0.62	19.3
B CELLS	NORMAL	-1.07	42.2
CD4 CELLS	NORMAL	-1.11	44.0
CD8 CELLS	NORMAL	-1.16	48.5
CHECK POINT	NORMAL	-1.03	38.2
CHEMOKINES	NORMAL	-0.95	39.9
CYTOKINES	NORMAL	-0.87	30.2
CYTOTOXICITY	NORMAL	-1.88	85.9
ENDOTHELIUM	NORMAL	-1.52	66.4
MACROPHAGES	NORMAL	-1.48	77.7
NK CELLS	NORMAL	-0.97	24.6
PBPC1	NORMAL	-1.19	63.9
PBPC2	NORMAL	-0.21	1.8
PBPC3	NORMAL	-0.29	3.4
PBPC4	NORMAL	-0.30	4.3
PBPC5	NORMAL	-0.27	3.1
PBPC6	NORMAL	-0.49	11.4
PBPC7	NORMAL	-0.44	7.2
PBPC8	NORMAL	1.63	80.0
PLASMA CELLS	NORMAL	-1.23	50.8
TFHC	NORMAL	-1.35	63.0
TGFB Path	NORMAL	-0.87	30.6
TH1	NORMAL	-1.68	90.7
TH17	NORMAL	-1.19	51.0
TH2	NORMAL	-1.74	73.3
TNF Path	NORMAL	-1.53	74.4
TUBULES	NORMAL	1.08	45.8
TYPE1 INTERFERON	NORMAL	-1.45	66.9
TYPE2 INTERFERON	NORMAL	-1.62	98.2
UPC1	NORMAL	-1.04	69.6
UPC2	NORMAL	-0.13	0.9
UPC3	NORMAL	-0.69	20.3
UPC4	NORMAL	-0.42	8.0
UPC5	NORMAL	1.64	133.9
UPC6	NORMAL	-0.21	2.1
UPC7	NORMAL	-0.05	0.3
