# Supplementary material for: In-silico performance, validation, and modeling of the Nanostring Banff Human Organ transplant gene panel using archival data from human kidney transplants
Source: BMC Med Genomics. 2021 Mar 19;14:86. doi: 10.1186/s12920-021-00891-5 (PMC7977303; doi:10.1186/s12920-021-00891-5)
Supplement: Supplementary file 4 — Additional file 4. Supplementary Table 4. Error Matrices from Classification of Bayesian Networks [file 12920_2021_891_MOESM4_ESM.pdf]

SUPPLEMENTAL TABLE 4 BAYESIAN NETWORK

A

DIAGNOSIS ERROR MATRIX

DIAGNOSIS (N)	ABMR (105)	AKI (28)	MIXED (28)	NOREJECTION (514)	NORMAL (22)	TCMR (67)	Total	Errors	% Errors
ABMR (103)	32	0	5	63	0	3	103	71	68.9%
AKI (87)	3	23	0	52	6	3	87	64	73.6%
MIXED (93)	16	0	12	38	0	27	93	81	87.1%
NOREJECTION (284)	35	2	2	232	3	10	284	52	18.3%
NORMAL (96)	5	3	0	75	13	0	96	83	86.5%
TCMR (101)	14	0	9	54	0	24	101	77	76.2%
TOTAL	105	28	28	514	22	67	764	428	56.0%

RESAMPLING K FOLD = 10	
Mean ROC Index	76.9%
Overall Log-Loss	3.8

B

CLUSTER ERROR MATRIX

CLUSTER (N)	1 (33)	2 (44)	3 (170)	4 (54)	5 (103)	6 (23)	7 (130)	8 (87)	9 (120)	TOTAL	ERRORS	% ERRORS
1 (2)	0	0	0	0	1	0	0	0	1	2	2	100.0%
2 (36)	1	25	1	0	6	1	2	0	0	36	11	30.6%
3 (251)	15	2	137	0	15	12	13	21	36	251	114	45.4%
4 (54)	0	1	0	45	4	0	2	2	0	54	7	13.0%
5 (78)	4	2	0	1	62	0	6	3	0	78	16	20.5%
6 (6)	0	0	0	0	0	6	0	0	0	6	0	0.0%
7 (127)	0	5	5	8	6	0	95	3	5	127	32	25.2%
8 (86)	9	0	4	0	8	0	7	55	3	86	31	36.0%
9 (124)	4	9	23	0	1	4	5	3	75	124	49	39.5%
TOTAL	33	44	170	54	103	23	130	87	120	764	262	34.3%

RESAMPLING K FOLD = 10	
Mean ROC Index	90.5%
Overall Log-Loss	1.0240
