# Supplementary material for: In-silico performance, validation, and modeling of the Nanostring Banff Human Organ transplant gene panel using archival data from human kidney transplants
Source: BMC Med Genomics. 2021 Mar 19;14:86. doi: 10.1186/s12920-021-00891-5 (PMC7977303; doi:10.1186/s12920-021-00891-5)
Supplement: Supplementary file 5 — Additional file 5. Figure 1 Kernel Density Estimates of Principal Components (JMP Genomics9.2/Distribution). 1A. Kernel density estimates, all PCs. 1B-1D. Kernel density estimates of PBCP (1B), CPPC (1C), and UPC (1D) by Diagnosis. [file 12920_2021_891_MOESM5_ESM.pdf]

SUPPLEMENTAL FIGURE 1

1A

Kernel Density Estimates All Principals Components

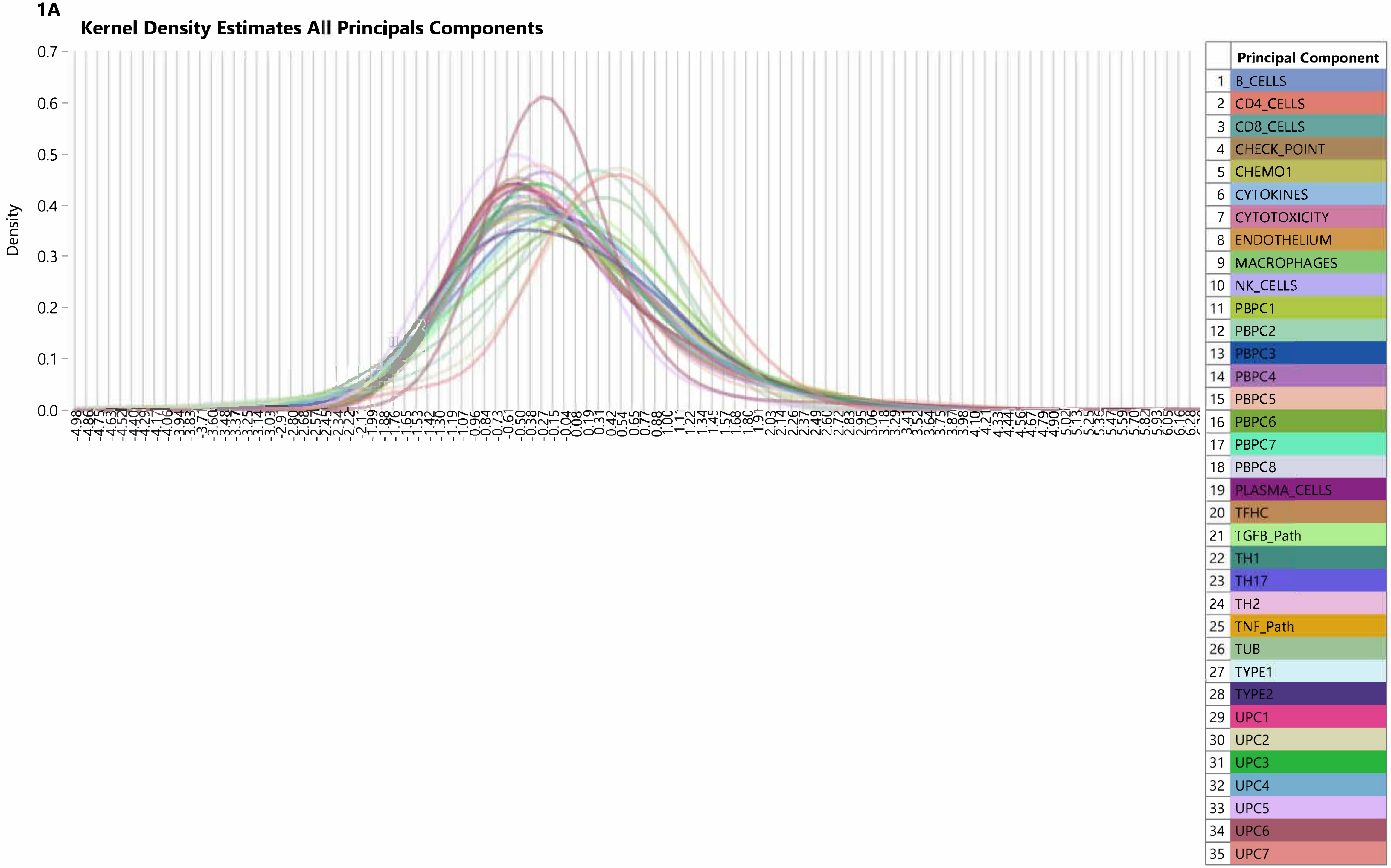

1B

Kernel Density Estimates Pathologically Based Principal Components PBPC

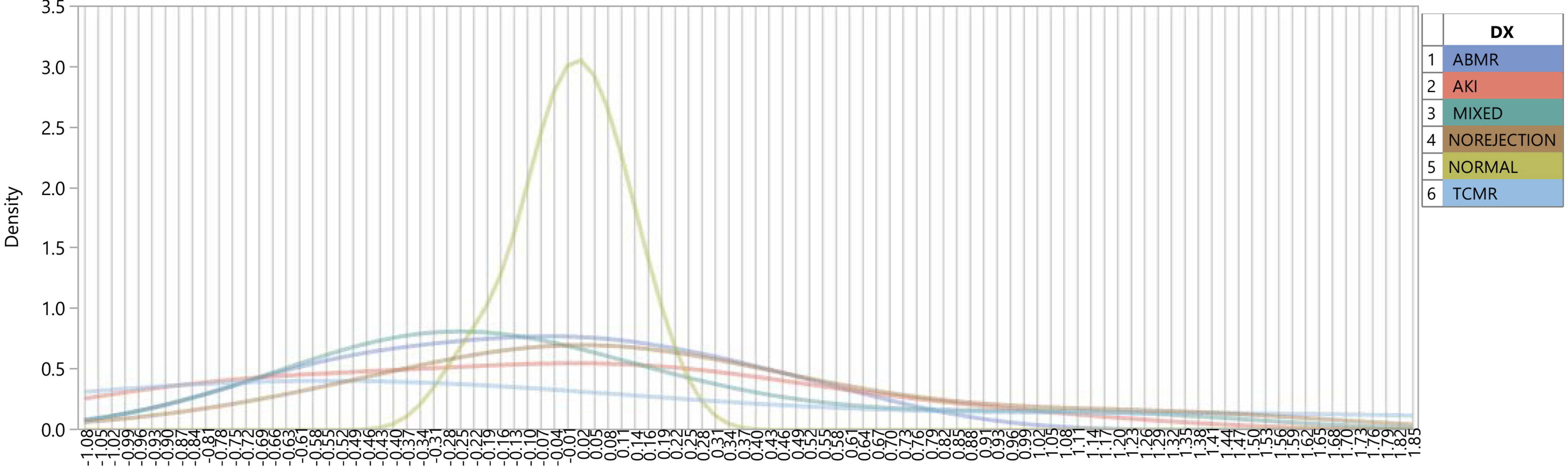

1C

Kernel Density Estimates Cell Pathways Principal Components CPPC

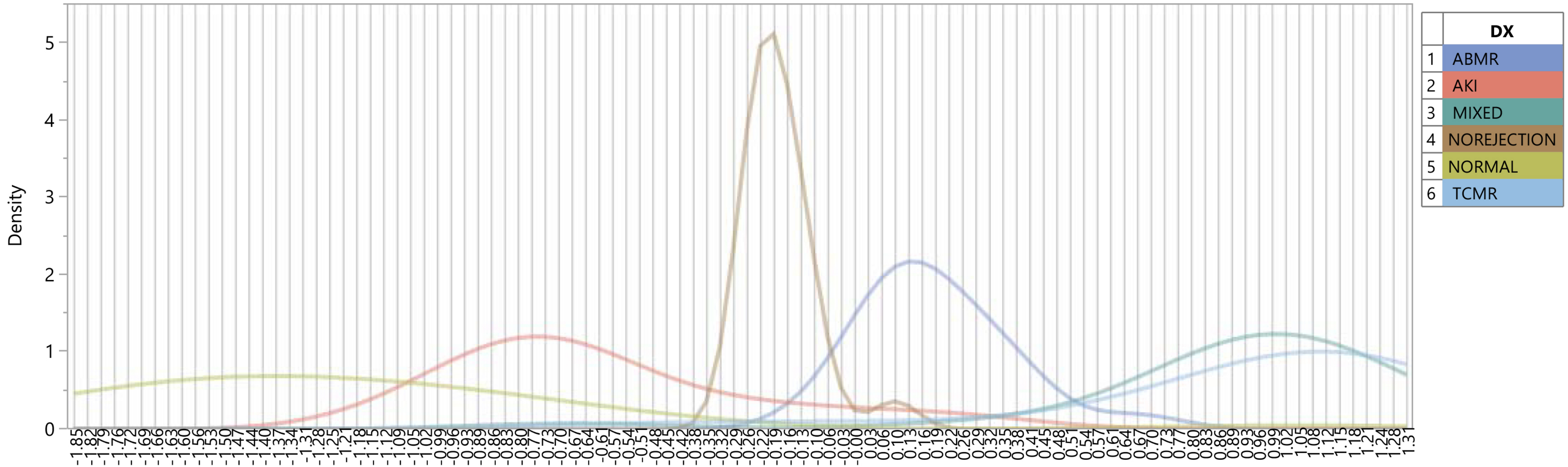

1D

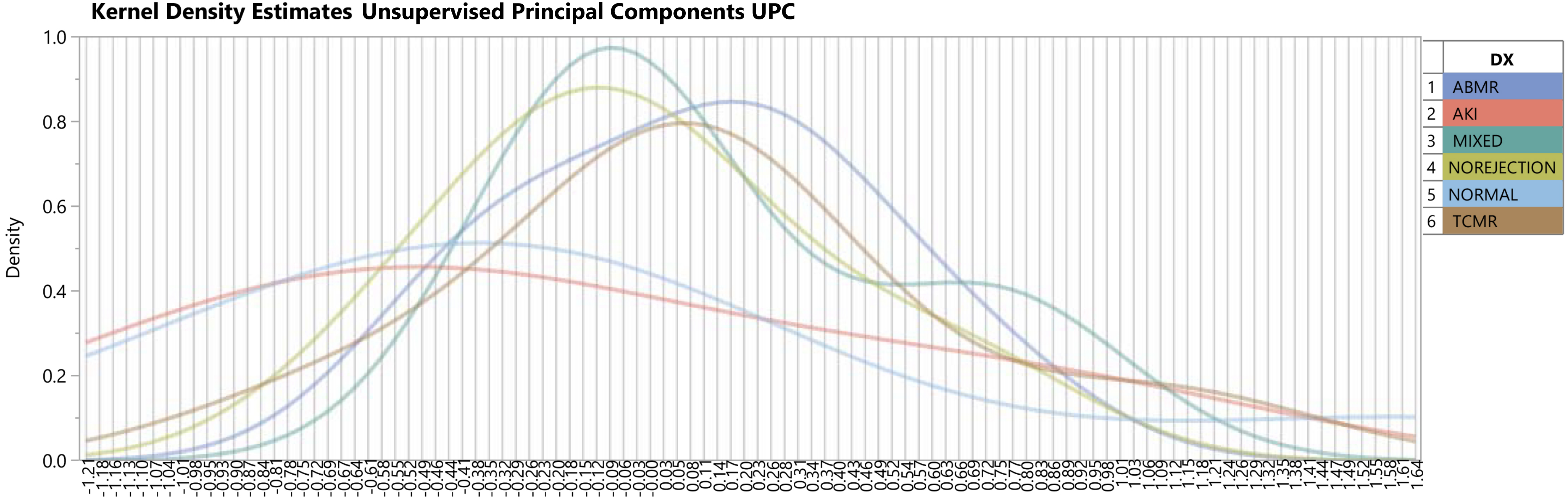
Kernel Density Estimates Unsupervised Principal Components UPC
Density
1.0
0.8
0.6
0.4
0.2
0.0
DX
1 ABMR
2 AKI
3 MIXED
4 NOREJECTION
5 NORMAL
6 TCMR
