# Supplementary figures and images for: In-silico performance, validation, and modeling of the Nanostring Banff Human Organ transplant gene panel using archival data from human kidney transplants
Source: BMC Med Genomics. 2021 Mar 19;14:86. doi: 10.1186/s12920-021-00891-5 (PMC7977303; doi:10.1186/s12920-021-00891-5)

SUPPLEMENTAL FIGURE 2

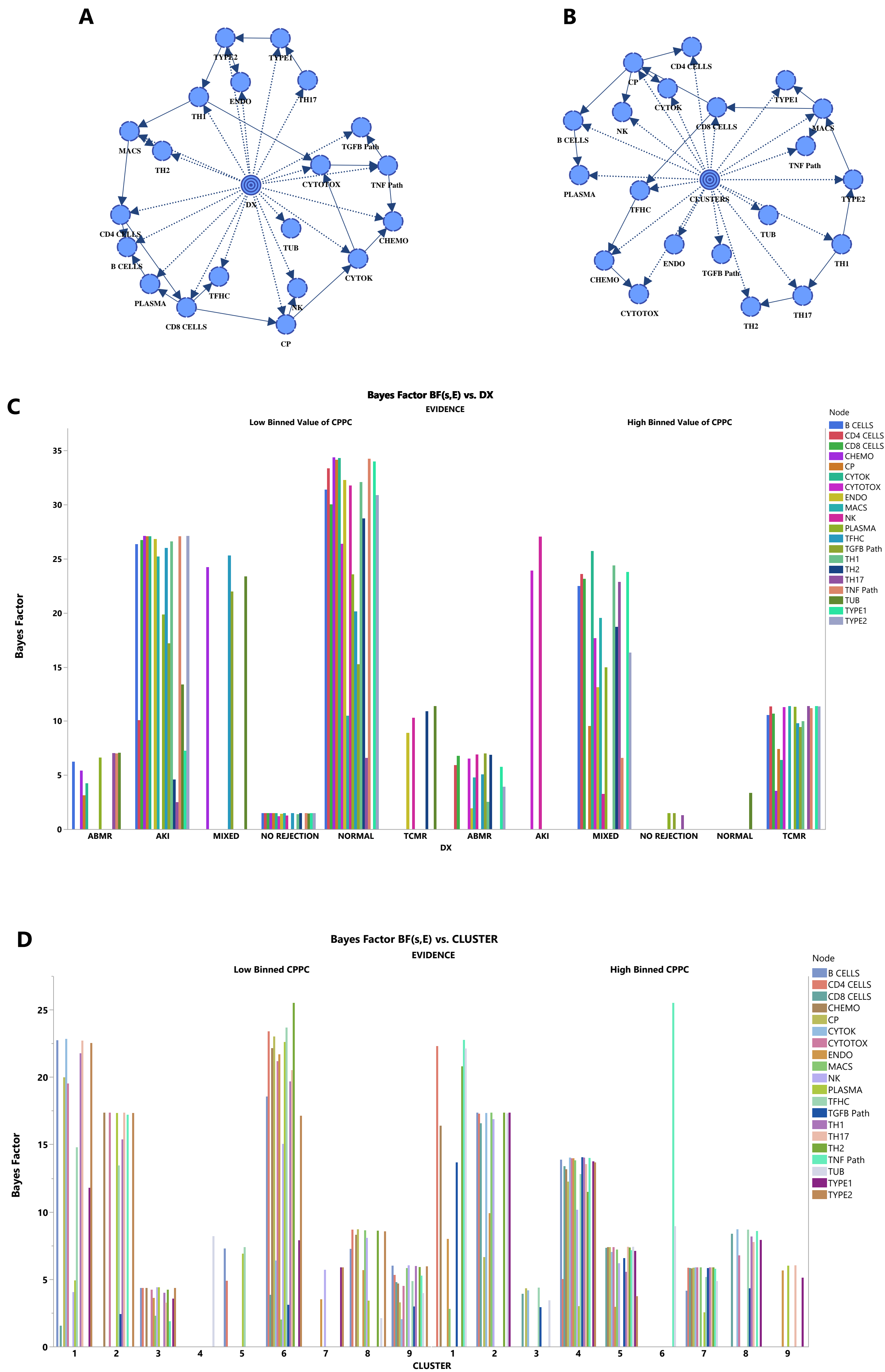

Supplement: Supplementary file 6 — Additional file 6. Figure 2. Graphical representation of Bayesian Networks for DX (A) and Cluster (B). Bayesia Lab 9.4. Discretization: Perturbed Tree, Bins = 2; Supervised Learning = Tree Augmented Naïve Bayes, both determined empirically by minimal descriptive length. Bargraphs of the Bayes Factors for DX (C) and Clusters (D) following calculation for Kulback-Leibler divergence [file 12920_2021_891_MOESM6_ESM.pdf]
